# Methodological developments in searching for studies for systematic reviews: past, present and future?

**DOI:** 10.1186/2046-4053-2-78

**Published:** 2013-09-25

**Authors:** Carol Lefebvre, Julie Glanville, L Susan Wieland, Bernadette Coles, Alison L Weightman

**Affiliations:** 1 Lefebvre Associates Ltd, Oxford, UK; 2 York Health Economics Consortium, University of York, York, UK; 3 Brown University, Providence, RI, USA; 4 Cardiff University, Cardiff, UK

**Keywords:** Cochrane Collaboration, Information and communication technologies, Information retrieval, Quality, Searching, Standards, Training materials, Trials Search Co-ordinators, Unpublished studies, Updating reviews

## Abstract

The Cochrane Collaboration was established in 1993, following the opening of the UK Cochrane Centre in 1992, at a time when searching for studies for inclusion in systematic reviews was not well-developed. Review authors largely conducted their own searches or depended on medical librarians, who often possessed limited awareness and experience of systematic reviews. Guidance on the conduct and reporting of searches was limited. When work began to identify reports of randomized controlled trials (RCTs) for inclusion in Cochrane Reviews in 1992, there were only approximately 20,000 reports indexed as RCTs in MEDLINE and none indexed as RCTs in Embase. No search filters had been developed with the aim of identifying all RCTs in MEDLINE or other major databases. This presented The Cochrane Collaboration with a considerable challenge in identifying relevant studies.

Over time, the number of studies indexed as RCTs in the major databases has grown considerably and the Cochrane Central Register of Controlled Trials (CENTRAL) has become the best single source of published controlled trials, with approximately 700,000 records, including records identified by the Collaboration from Embase and MEDLINE. Search filters for various study types, including systematic reviews and the Cochrane Highly Sensitive Search Strategies for RCTs, have been developed. There have been considerable advances in the evidence base for methodological aspects of information retrieval. *The Cochrane Handbook for Systematic Reviews of Interventions* now provides detailed guidance on the conduct and reporting of searches. Initiatives across The Cochrane Collaboration to improve the quality *inter alia* of information retrieval include: the recently introduced Methodological Expectations for Cochrane Intervention Reviews (MECIR) programme, which stipulates 'mandatory’ and 'highly desirable’ standards for various aspects of review conduct and reporting including searching, the development of Standard Training Materials for Cochrane Reviews and work on peer review of electronic search strategies. Almost all Cochrane Review Groups and some Cochrane Centres and Fields now have a Trials Search Co-ordinator responsible for study identification and medical librarians and other information specialists are increasingly experienced in searching for studies for systematic reviews.

Prospective registration of clinical trials is increasing and searching trials registers is now mandatory for Cochrane Reviews, where relevant. Portals such as the WHO International Clinical Trials Registry Platform (ICTRP) are likely to become increasingly attractive, given concerns about the number of trials which may not be registered and/or published. The importance of access to information from regulatory and reimbursement agencies is likely to increase. Cross-database searching, gateways or portals and improved access to full-text databases will impact on how searches are conducted and reported, as will services such as Google Scholar, Scopus and Web of Science. Technologies such as textual analysis, semantic analysis, text mining and data linkage will have a major impact on the search process but efficient and effective updating of reviews may remain a challenge.

In twenty years’ time, we envisage that the impact of universal social networking, as well as national and international legislation, will mean that all trials involving humans will be registered at inception and detailed trial results will be routinely available to all. Challenges will remain, however, to ensure the discoverability of relevant information in diverse and often complex sources and the availability of metadata to provide the most efficient access to information. We envisage an ongoing role for information professionals as experts in identifying new resources, researching efficient ways to link or mine them for relevant data and managing their content for the efficient production of systematic reviews.

## The situation in 1992/1993

The Cochrane Collaboration was established in 1993, with roots in the opening of the UK Cochrane Centre in October 1992. Other than the Information Specialist employed at the UK Cochrane Centre (CL), there were no Trials Search Co-ordinators (TSCs) employed within The Cochrane Collaboration in the early 1990s. The term TSC, now generally used across The Cochrane Collaboration for the information specialists who identify relevant studies for inclusion in Cochrane Reviews, did not exist. Authors relied on their own skills and expertise in searching to identify reports of studies or on the skills of medical librarians, who often had limited awareness of systematic reviews and the specific searching approaches required. There was little training for, or awareness amongst, medical librarians regarding the role, importance and complexity of systematic reviews and little focus on searching for adverse events, economic evaluations, non-randomized designs or qualitative studies. There was very limited guidance for the authors of Cochrane Reviews or Cochrane Review Group staff with respect to the conduct or reporting of searches.

When work began at the UK Cochrane Centre in 1992 to identify reports of randomized controlled trials (RCTs) for inclusion in Cochrane Reviews, approximately 20,000 reports were indexed as RCTs in MEDLINE. Randomized Controlled Trial had only been introduced into MEDLINE as a Publication Type term in 1991 and at that time there was no indexing term at all for quasi-randomized studies. In Embase, there were no indexing terms whatsoever for RCTs or quasi-randomized studies. No filters had been designed specifically to identify all RCTs in MEDLINE or any of the other major databases (irrespective of other study characteristics). This presented a tremendous challenge for a newly-established organization such as The Cochrane Collaboration in terms of efficient identification of relevant studies [1,2].

## Information retrieval methods: celebrating the first 20 years of The Cochrane Collaboration

In December 1992, a meeting was held with Elsevier which led to the introduction of RCT as an indexing term into Embase in 1993 [3] and a commitment by Elsevier to improve the indexing of clinical trials [4]. This was followed in December 1993 by a conference hosted by the US National Library of Medicine (NLM) which led to agreement to 're-tag’ RCTs in MEDLINE (the MEDLINE re-tagging project). This project resulted in more than 125,000 reports of randomized and quasi-randomized trials, not already indexed as such in MEDLINE, being identified, re-tagged in MEDLINE and included in the Cochrane Central Register of Controlled Trials (CENTRAL).

In 1995, a new indexing term for quasi-randomized trials (Controlled Clinical Trial) was introduced into MeSH [5]. The Cochrane Central Register of Controlled Trials (CENTRAL) was launched in 1996 (under its original name of The Cochrane Controlled Trials Register), as part of the newly published *Cochrane Library*[6]. CENTRAL was referred to in its early days as 'likely to be the best single source of published trials for inclusion in systematic reviews and meta-analyses’ [7]. In 1996, Elsevier agreed that reports of trials identified from Embase could be included in CENTRAL (the Embase project). CENTRAL developed rapidly over the next few years [6]. The contributions of the MEDLINE re-tagging project [6,8] and the Embase project [3] now form the basis of CENTRAL, which is the single largest source of reports of trials, with 700,000 records drawn from MEDLINE, Embase, Cochrane groups and other sources [9].

Considerable progress has been made in searching across a range of areas important to the systematic review process. Some examples include the identification of information on adverse effects of interventions [10-14]; diagnostic test studies [15-17]; economic evaluation [18-21]; studies of prognosis [22-24] and causation [24-27]; non-randomized studies related to interventions [28] and qualitative studies [29-33]. Progress has also been made in the identification of systematic reviews, as sources of potential studies [34-37].

With respect to RCTs, filters aimed at identifying all RCTs in MEDLINE, irrespective of other study characteristics, began to be developed by members of The Cochrane Collaboration in the early 1990s [2]. They were revised using objective methods of search strategy design (textual analysis) in 2006 [38] and subsequently kept up to date in the 'Searching for Studies’ chapter of *The Cochrane Handbook for Systematic Reviews of Interventions*[39].

The proliferation of search filters across a range of methodological areas, and their ease of access through incorporation in services such as Ovid and PubMed, has led to the development of the InterTASC Information Specialists’ Sub-Group (ISSG) Search Filter Resource. This offers critical appraisals and summaries of search filters together with references to comparative testing data [40,41]. Given the growing interest in identifying information beyond particular types of study, such as age groups, geographic areas and ethnic groups, the ISSG Search Filter Resource has recently been expanded to incorporate these topics. Examination of the conduct and reporting of searches for Cochrane Reviews [42,43] has led to structured approaches to peer review of search strategies (for example, the Peer Review of Electronic Search Strategies (PRESS) checklist) [44-47].

In addition to the advances in the identification of studies from bibliographic databases outlined above, methodological work has been undertaken on the value of searching the 'grey literature’ which has been defined as 'information produced and distributed at all levels by government, academics, business and industry in electronic and print formats not controlled by commercial publishing i.e. where publishing is not the primary activity of the producing body’ [48,49]. Related research has assessed the value of handsearching for trials [50-52] and examined the characteristics associated with full publication of meeting abstracts [53]. Alternative search techniques such as 'pearl-growing’/'snowballing’ from known key references and checking reference lists have also been explored [54,55].

Keeping up-to-date in methodological advances has been facilitated by the advent and development of the Cochrane Methodology Register, published in *The Cochrane Library*. Updating of this resource is currently on hold pending decisions regarding its future within The Cochrane Collaboration. The recently-launched Summarized Research in Information Retrieval for Health Technology Assessment (SuRe Info) provides research-based information regarding the latest developments in the information retrieval aspects of producing systematic reviews and health technology assessments [56]. It provides critical appraisals and summaries of current methods papers and general overviews of the state of the evidence across a range of topics relevant to information retrieval for systematic reviews.

The guidance in Chapter 6 of *The Cochrane Handbook for Systematic Reviews of Interventions* entitled 'Searching for Studies’ started life in 1994 as an internal Cochrane document produced by Kay Dickersin and Carol Lefebvre entitled 'Establishing and Maintaining Registers of RCTs’. The document provided limited guidance with respect to the conduct and reporting of searches. This subsequently became incorporated into the *Handbook* and now provides detailed guidance for authors of Cochrane Reviews and Cochrane Review Group staff, including TSCs, Managing Editors, Co-ordinating Editors and Editors [39]. It is also used by other evidence synthesis organizations and provided a model for the Campbell Collaboration’s Guide to Information Retrieval for Campbell Systematic Reviews [57]. The *Handbook* is revised and updated in consultation with the information retrieval community of The Cochrane Collaboration, that is, the Cochrane Information Retrieval Methods Group and TSCs. Standard Training Materials have been produced and have been updated in the light of the standards recently introduced under the Cochrane Methodological Expectations of Cochrane Intervention Reviews programme (MECIR) [58].

In 2013, almost all Cochrane Review Groups and some Cochrane Centres and Fields have a dedicated TSC – usually a qualified librarian/information specialist with experience of searching the medical literature. These TSCs carry out a vital role in study identification within their respective groups, although the nature of their contributions varies considerably according to resources and other factors. There is also far greater awareness amongst medical librarians and other information specialists regarding the role of systematic reviews and how to search for studies for inclusion in systematic reviews.

## Focus on the future: the next five to ten years

### Information and data sources

Prospective registration of clinical trials, already encouraged by initiatives including that of the International Committee of Medical Journal Editors (ICMJE) [59], should increase as a result of pressure from a range of consumer, legal and professional sources [60,61]. The use of data from trials registers within Cochrane Reviews will grow as a result of MECIR, which requires that trials registers and repositories of results, where relevant to the topic, be searched through ClinicalTrials.gov, the ICTRP and other sources as appropriate [58].

The challenges around identifying data from unpublished studies will be better understood and become more quantifiable as a result of research in this area, such as the project on searching for unpublished trials funded in 2011 by the Cochrane Methods Infrastructure Funding initiative [62]. Despite the introduction of new registers such as the EU Clinical Trials Register, the single portal approach offered by the ICTRP is likely to become increasingly attractive as a means to search across a range of registers from one site [63]. There is already concern about the number of trials which may not be registered and/or published and which prove difficult to retrieve, as in the case of Tamiflu [64,65]. Identifying unpublished data or trial reports may well focus on increased efforts to utilize sources such as regulatory agency data (for example, the European Medicines Agency, the US Food and Drug Administration (FDA)) and reports from agencies such as the National Institute for Health and Care Excellence (NICE) in the UK. There is also likely to be increased pressure for access to clinical study reports produced by manufacturers despite resistance from certain manufacturers [66,67]. Obtaining data from clinical study reports will have a considerable impact on the production of systematic reviews due to the extensive nature of the documents and lack of standardization across manufacturers [68]. The Cochrane Register of Studies is an internal data repository and data management tool within The Cochrane Collaboration. It will be further developed and integrated with CENTRAL, to serve as a 'meta-register’ or repository for Specialized Registers (registers of studies and/or reports of studies relevant to a specific Cochrane group) and all other trial records submitted by Cochrane groups. This integration will introduce time efficiencies in identifying which reports are associated with which studies.

Increasingly, search interfaces (as can be seen currently with Ovid and Web of Knowledge) will offer cross-database searching options. These have the potential to improve the efficiency of database searching by reducing redundancy associated with searching multiple databases separately (and the need for de-duplication). Reassurances will be required, however, that searches are being conducted and interpreted correctly in the individual databases, that is, that the results retrieved by a cross-database search are equivalent to the results of searching the databases individually. The availability of full-text databases of journal articles and other documents will also create new opportunities to access larger quantities of text for searching than has been the case previously. The increasing availability of gateways, or portals, such as Science.gov, which offers access to science information and research results from a number of US federal agencies, albeit via a relatively unsophisticated search interface, will enable wider searching of the grey literature [69]. Options for incorporating citation searching within reviews are increasing through resources such as Scopus and Web of Science and are freely accessible via Google Scholar. The ability to download records from the latter increases its attractiveness as a tool for systematic reviewers, despite the current lack of sophistication in the search interface.

### Search strategies and techniques

Following search approaches used within public health, such as for NICE guidance [70] and diagnostic test accuracy reviews [71], we may see more use of multi-faceted search techniques using several combinations of concepts to capture a review topic, rather than single PICO-style (Population, Intervention, Comparison, Outcome) search strategies or variants of PICO, particularly with more complex review questions. Search strategies may increasingly be developed using textual analysis techniques for individual subject search strategies [72]. These approaches will use freely accessible off-the-shelf software such as PubMed PubReMiner or commercially available statistical software packages such as SimStat/WordStat to identify highly-discriminating search terms from pre-defined sets of relevant records.

Use of semantic analysis or text mining software will increase, in the place of, or as an adjunct to, Boolean searching and/or textual analysis, and also in the context of the design of methodological search filters [73]. This will mean that searches will be conducted based on the meaning of words and concepts within a set of records, rather than simply the presence of these terms or concepts. Semantic analysis will help with complex review questions or 'hard to capture’ topics, such as those addressed in public health. The semantic analysis approach may be used to interrogate large result sets to retrieve records likely to be relevant to a query in decreasing order of probability of relevance [74]. This may involve two-step searching approaches (gathering search results using very sensitive Boolean searches then interrogating those results using semantic analysis software) or semantic analysis may be built into internet search portals. With growing use of data linkage it will become increasingly possible to mine the internet from key references to find related and citing works. The challenge currently, however, is in searching ever richer resources with interfaces which are far from sophisticated and which do not facilitate complex searches or offer search facilities, such as saving searches or downloading records.

These developments will present challenges for the peer review of the search process including search strategies and the current Cochrane pilot study on peer review may require rapid evolution. The impact of the increasingly diverse options for trial discovery via data linkage and the growth in portals will have considerable impact on reporting the search process with respect to transparency and reproducibility. Documentation will become increasingly crucial as the internet becomes ever more organic. The requirement to demonstrate search effectiveness will continue but may become more complex to achieve. Reference management software will become standard, especially for de-duplication of multiple database searches and massive result sets arising from text mining approaches, but may become merged with semantic analysis software.

Self-audit of the search process and search strategies will become more common as awareness of, and familiarity with, techniques such as capture-recapture (that is, estimating the number of relevant records by conducting two sample searches and comparing the number of relevant records identified in the first search that were then also identified by the second search) [75,76] and relative recall [77] grows. Self-audit will also be influenced by an increase in more formal audit approaches undertaken by the commissioners or funders of reviews. Techniques such as relative recall will be used to make judgements about which databases need to be searched and how comprehensive the search strategies need to be for each database, to help address the perennial question of 'when is enough enough’ [78-80]. Concerns over the generalizability and reliability of these approaches, however, are likely to remain.

### Updating reviews and evolution of information retrieval methods

The challenges associated with updating reviews may be mitigated to some extent by techniques such as searches based on previously 'included’ studies (for example, citation searches of the 'largest’/'newest’ studies [81]) and 'horizon-scanning’ for 'trials that would make a difference’ [82]. Increased data linkage may make the updating process more streamlined and current. The further development of trials registers and increased pressure for trial registration should also make it easier to identify 'important’ trials as they reach completion.

Information retrieval methods for Cochrane Reviews will continue to benefit from research conducted outside of The Cochrane Collaboration by organizations involved in systematic reviews, meta-analyses, health technology assessment and other evidence syntheses and will be informed by processes and standards produced by other organizations such as the Agency for Healthcare Research and Quality (AHRQ) [83], the Centre for Reviews and Dissemination [84], the US Institute of Medicine [85] and the National Institute for Health and Care Excellence (NICE) [70] as well as by initiatives for assessing methodological quality or standardizing reporting such as AMSTAR (A Measurement Tool to Assess Systematic Reviews) [86,87], PRISMA (Preferred Reporting Items for Systematic Reviews and Meta-Analyses [88,89] and CONSORT (Consolidated Standards of Reporting Trials) [90,91]. In turn, the major revision of *The Cochrane Handbook for Systematic Reviews of Interventions*, scheduled for publication in 2014, has the potential to continue to have considerable influence both within and beyond The Cochrane Collaboration. In future, emerging technologies will enable the information in the *Handbook* to be presented and utilized in more imaginative and accessible ways.

The quality of information retrieval aspects of Cochrane Reviews will be enhanced by further implementation, expansion and revision of the MECIR standards [58], in the light of feedback based on early implementation, audit results, the initiation of standards for review protocols and updates and other quality improvement measures. As a result of MECIR standards and other guidance, such as that developed by the US Institute of Medicine [85], multidisciplinary working involving a librarian or other information specialist trained in performing systematic reviews to plan the search process and the search strategies is likely to become more prevalent, along with the use of an independent librarian or other information specialist for peer review of the study identification elements of reviews.

This greater involvement is being supported by the increased training which is available for librarians and information specialists in a wide range of aspects of information retrieval in the context of evidence synthesis.

## Focus on the future: 2033 and beyond

In making any assessments as to the possible situation in twenty years’ time, we should be mindful of the words attributed to Niels Bohr, the Danish physicist (1885–1962): 'Prediction is very difficult, especially about the future’ [92]. In twenty years’ time, we envisage that universal social networking (or its successors) as well as national and international legislation will mean that all trials involving humans will be registered at inception. In addition, details of ongoing and completed trials will be accessible to all, irrespective of whether or not they have been published in the scientific literature, in a manner suitable for synthesis in systematic reviews and for other purposes. Registration of trials will become universal, in part, because information about trials will be broadcast by active trial participants who will publicize their experiences. Some compromise between commercial interests and public interests will have been reached so that far more detailed trial results will be available than we see at present, perhaps held in a common format in a single international clinical trials results register. This would build on the progress already made in creating and developing ClinicalTrials.gov [93] and the ICTRP [94]. The ClinicalTrials.gov dataset is already being used by The Cochrane Collaboration in the Cochrane Register of Studies and systems are being developed to add value to ClinicalTrials.gov, such as by downloading study results into a spreadsheet format ready for analysis [95]. All data from clinical trials required for systematic reviews and meta-analyses will be available in a single international clinical trials data repository, building on the progress already made in creating and developing the Systematic Review Data Repository (SRDR) [96]. Challenges will remain, however, in ensuring the discoverability of relevant information in these diverse and often complex sources and in developing the metadata needed to provide the most efficient access to information to answer specific questions reliably.

## Conclusions

Considerable progress has been made in the field of information retrieval within the context of systematic reviews over the last twenty years, as outlined above. There will, however, be many challenges as well as opportunities in the years ahead. We envisage that, in twenty years’ time, there will still be a role for experts in identifying new resources, researching efficient ways to link or mine them for relevant data and managing their content for the efficient production of systematic reviews. Whether these experts will be referred to as Trials Search Co-ordinators, Information Specialists or something else entirely in 2033, remains to be seen.

## Abbreviations

AHRQ: US Agency for Healthcare Research & Quality; AMSTAR: a measurement tool to assess systematic reviews; CENTRAL: Cochrane Central Register of Controlled Trials; CONSORT: CONsolidated Standards Of Reporting Trials; CRD: UK Centre for Reviews and Dissemination; EU: European Union; FDA: US Food and Drug Administration; ICTRP: WHO International Clinical Trials Registry Platform; MECIR: 'Methodological Expectations of Cochrane Intervention Reviews; MeSH: Medical Subject Heading; NICE: UK National Institute for Health and Care Excellence; NLM: US National Library of Medicine; PICO: 'Population, Intervention, Comparison, Outcome; PRESS: Peer Review of Electronic Search Strategies checklist; PRISMA: 'Preferred Reporting Items for Systematic Reviews and Meta-Analyses; RCT: Randomized controlled trial; SRDR: Systematic Review Data Repository; SuRe Info: 'Summarized Research in Information retrieval for health technology assessment; TSC: Trials Search Co-ordinator; WHO: World Health Organization.

## Competing interests

CL declares that, within the past five years, she has received reimbursements from the publishers of *The Cochrane Library* (John Wiley and Sons) towards travel expenses to attend conferences and deliver presentations about *The Cochrane Library*. She has not received any fees, other funding, or salary from John Wiley and Sons. She declares that, as an independent information specialist, she derives income from consultancy and teaching in information retrieval for evidence synthesis, including some of the topics addressed in this paper. Whilst employed at the UK Cochrane Centre, she was responsible for projects that contributed a significant proportion of the records to the Cochrane Central Register of Controlled Trials (CENTRAL). JG declares that she has received funding from The Cochrane Collaboration as a member of two consortia. As part of one consortium she managed the production of an annotated bibliography of research on identifying unpublished study data and in the second consortium she is managing the production of a search filter to identify reports of RCTs in Embase. She has not received any fees, other funding, or salary from The Cochrane Collaboration or their publishers, John Wiley and Sons. She derives income from consultancy and teaching in systematic reviewing and information retrieval for evidence synthesis, including some of the topics addressed in this paper. LSW declares that, whilst she was employed at the US Cochrane Center, she co-ordinated the project for re-tagging MEDLINE records and processed Cochrane handsearch records and Specialized Register records, which constituted a significant proportion of the records included in the Cochrane Central Register of Controlled Trials (CENTRAL). BC and AW declare that they have no competing interests.

## Authors’ contributions

CL was responsible for the conception and design of the paper; drafted the original outline of the paper; drafted the sections on the past and the present and revised the paper critically for important intellectual content. JG approved the original outline of the paper; drafted the sections on the future and revised the paper critically for important intellectual content. LSW approved the original outline of the paper; revised the paper critically for important intellectual content and identified relevant references. BC approved the original outline of the paper and created the EndNote Library and the Bibliography. AW approved the original outline of the paper and revised the paper critically for important intellectual content. All authors read and approved the final manuscript.

## Authors’ information

CL is an independent information specialist. She was a founding member of the UK Cochrane Centre (UKCC), where she was employed as the Senior Information Specialist from its inception, in 1992, until June 2012. She has an MSc in Library and Information Studies. She is a founding Co-Convenor of the Cochrane Information Retrieval Methods Group and a member of the Cochrane Methods Executive. She was a member of the Trials Search Co-ordinators Executive until 2012. JG is an Associate Director of York Health Economics Consortium at the University of York, York, UK. She has worked on systematic reviews since 1993 when she was one of the founding members of the Centre for Reviews and Dissemination, the sister organization to the UK Cochrane Centre. She has a Postgraduate Diploma in Librarianship and Information Studies and an MSc in Information Processing. She is a Co-Convenor of the Cochrane Information Retrieval Methods Group, has previously chaired the Cochrane Library Users’ Group and has provided extensive training in the use of *The Cochrane Library* over many years. She is a co-author of Cochrane Reviews and a peer reviewer for Cochrane Diagnostic Test Accuracy reviews. LSW is a Research Associate at the Center for Evidence-Based Medicine, Brown University, RI, US. She has a PhD in Epidemiology. She worked at the New England Cochrane Center, Providence Office in 2001–2002 and the US Cochrane Center from 2003–2005. She is the TSC for the Cochrane Complementary Medicine Field. BC has worked for Cardiff University since 1987 and is Site Librarian at the Cancer Research Wales Library in Cardiff, UK. She has an MSc in Health Information Management and became involved in systematic reviewing when the Cochrane Prostatic Diseases and Urological Cancers Group was established in 1998. She has recently been appointed as an Assistant TSC for this Group. She has been the Co-ordinator of the Cochrane Information Retrieval Methods Group since 2006. She is a co-author of 10 Cochrane Reviews and teaches a wide variety of courses for National Health Service staff in the UK. AW is the Associate Director for Research & Academic Engagement, Information Services and Director of the Support Unit for Research Evidence (SURE) at Cardiff University, UK. She has a Postgraduate Diploma in Librarianship, an MA in Library and Information Science and a PhD in Microbiology. She is a Co-Convenor of the Cochrane Information Retrieval Methods Group and a Cochrane Review author. She has more than 15 years' experience as a systematic reviewer and a particular interest and expertise in reviewing and developing systematic review techniques for complex public health topics.
